# COVID-19 and dentistry in sub-Saharan Africa: an urgent need to strengthen preventive measures in oral health care settings

**DOI:** 10.11604/pamj.supp.2020.35.2.23459

**Published:** 2020-05-20

**Authors:** William Ndjidda Bakari, Célestin Danwang, Mazou Ngou Temgoua

**Affiliations:** 1 Clinical Periodontology Training Program, Cheikh Anta Diop University, Dakar, Senegal; 2 Epidemiology and Biostatistic Unit, Institute of Experimental and Clinical Research, Catholic University Louvain, Brussels, Belgium; 3 Department of Internal Medicine, Faculty of Medicine and Biomedical Sciences, University of Yaoundé 1, Yaoundé, Cameroon

**Keywords:** COVID-19, dentistry, sub-Saharan Africa

## To the editors of the Pan African Medical Journal

In December 2019, a new virus causing pneumonia called Severe Acute Respiratory Syndrom Coronavirus 2 (SARS-COV 2) appeared in the city of Wuhan in China [1]. Since then, the disease named COVID-19 has spread around the world and was declared a pandemic on March 11, 2020 by the World Health Organization. As of May 10, 2020, the number of cases is estimated to 3,917,366, with 274,361 associated deaths. Even if the number of cases increased gradually, Africa still remains the least affected continent with 42,626 cases to date and 1,369 deaths [2]. SARS-COV 2 is highly contagious, easily transmitted from person to person through respiratory droplets from coughing, sneezing or by direct contact of contaminated hands (with the virus present on an inert surface) with the oral, nasal or ocular mucosa [3]. Transmission through saliva has been reported [4]. Symptomatic, asymptomatic as well as persons in the incubation phase of the infection, can be the vector of transmission of the disease to healthy individuals [3,5]. Thus, one of the global response strategies to curb down the burden of the disease has been to control the infection sources in order to reduce virus transmission risk.

Early diagnosis based on cases definition, isolation and supportive therapy in severe forms were adopted [1]. In the absence of a specific treatment and an effective vaccine, the resurgence of cases is a worrying problem for all health systems around the world. In Africa, where employment is largely based on the informal sector, the feasibility of total containment of the population to restrain the epidemic has been rapidly overtaken. Screening, early diagnosis and follow-up of contact cases as well as the application of distancing measures remain the actions applied in many African countries to limit the transmission of this virus in the community [6]. Dental professionals remain particularly exposed to the virus among healthcare professionals. Indeed, the quality of acts and procedures in dental practice increases the risk of contamination [7]. This contamination’s risk may arise from the direct face-to-face communication with patients; the use of ultrasound and high velocity rotating devices generating aerosols of saliva and/or blood; and sharp instruments handling [7]. Thus, very early in the epidemic, a restriction of oral care activity was requested [8].

Infection control and prevention of transmission, therefore, become a permanent challenge in the dental care and require additional resources especially in resources constraints setting such as sub-Saharan Africa. The following measures has been proposed to reduce the risk of transmission for dental health care worker: reorganization of the reception system with systematic temperature-taking of patients; a questionnaire (in order to stratify patients according to their risk of being infected) asking for the general health status, a history of travel to an epidemic zone in the previous 14 days and a close contact with an infected or suspected positive SRAS-COV 2 individual [9]. Furthermore, additional precautions in terms of personal protection (smock, goggles, face shields, N95 or FFP2 mask) must be systematically adopted for patient’s management during this period [6]. The limitation of dental care during this pandemic to emergency care remains to be evaluated in our daily practice.

While business support plans have been developed and include dental professionals in developed countries, in sub-Saharan Africa few efforts in this direction have been undertaken [10]. There is an urgent need to strengthen infection control and prevention measures in oral health care settings. In a context of limited resources and lacking or non-optimal amount of protective equipment, this equation becomes even more difficult to solve and raises fears that dental care units could become a real vector of infection transmission in communities. Awareness must therefore be raised among policy makers on the need to provide significant material support and additional training to dental professionals in order to ensure quality of care and to avoid transmission of the disease in this setting.

## Conclusion

The COVID-19 pandemic in sub-Saharan Africa brings up a big challenge to the continuity of oral health care provision. Adaptation and reshaping seem necessary to reduce the contamination’s risk for staff and patients. It would be important to support these professionals to maintain their activity while ensuring safety for patients and the community.

## Competing interests

The author declares no competing interests.
